# High Prevalence of Infertility among Women with Graves' Disease and Hashimoto's Thyroiditis

**DOI:** 10.1155/2014/982705

**Published:** 2014-02-11

**Authors:** Alessandra Quintino-Moro, Denise E. Zantut-Wittmann, Marcos Tambascia, Helymar da Costa Machado, Arlete Fernandes

**Affiliations:** ^1^Department of Obstetrics and Gynecology, School of Medical Sciences, University of Campinas, Rua Alexander Fleming, 101, Cidade Universitária, 13083-881 Campinas, SP, Brazil; ^2^Endocrinology Division of Department of Clinical Medicine, School of Medical Sciences, University of Campinas, Rua Tessália Vieira de Camargo, 126, Cidade Universitária, 13083-887 Campinas, SP, Brazil; ^3^Statistical Office, School of Medical Sciences, University of Campinas, Rua Tessália Vieira de Camargo, 126, Cidade Universitária, 13083-887 Campinas, SP, Brazil

## Abstract

*Objectives*. To evaluate the prevalence of infertility in women with Graves' disease (GD) or Hashimoto's thyroiditis (HT) and associated factors. *Material and Methods*. This cross-sectional study was conducted at the Endocrinology Clinic for Thyroid Autoimmune Diseases, with 193 women aged 18–50 years with GD and 66 women aged 18–60 years with HT. The women were interviewed to obtain data on their gynecological and obstetric history and family history of autoimmune diseases. Their medical records were reviewed to determine the characteristics of the disease and to confirm association with other autoimmune diseases. Infertility was defined as 12 months of unprotected sexual intercourse without conception. *Results*. The prevalence of infertility was 52.3% in GD and 47.0% in HT. Mean age at diagnosis was 36.5 years and 39.2 years, in GD and HT, respectively. The mean number of pregnancies was lower in women who were 35 years old or younger at diagnosis and was always lower following diagnosis of the disease, irrespective of age. The only variable associated with infertility was a shorter time of the disease in HT. *Conclusions*. The prevalence of infertility was high in women with GD and HT and affected the number of pregnancies in young women.

## 1. Introduction 

Infertility is defined as an inability to become pregnant following 12 months of unprotected sexual intercourse [1]. The prevalence of infertility is estimated at around 10–15% of couples, based on those seeking specific treatment; nevertheless, this prevalence differs between countries. As far as we know, two population-based studies about infertility prevalence were published. The first used data from four studies performed within the United States National Survey of Family Growth included women of 15–44 years of age living with a partner, not in use of contraceptive method and who had not conceived within the previous 12 months and showed a reduction in the estimated prevalence rates of infertility from 8.5% in 1982 to 7.4% in 2002 [2]. The other was a France-based study with a nationwide sample of families, identified 867 women of 18–44 years of age who reported having unprotected sexual intercourse with a male partner and who related the duration of exposure from the beginning of the unprotected period until the time of the survey. After 12 and 24 months of exposure, 24% and 11% of couples, respectively, had still not become pregnant [3].

Nevertheless, there are no data on the prevalence of infertility in specific groups of women with autoimmune diseases, particularly those diseases that occur during the reproductive years and affect the endocrine system. Of these, autoimmune thyroid dysfunction is the most common. Graves' disease (GD) affects around 1% of the population, while Hashimoto's thyroiditis (HT) affects around 3% [4]. These diseases consist of hyper- and hypothyroidism, both of which may interfere with the mechanisms of ovulation and with sex hormone metabolism, affecting fertility and, if pregnancy does occur, increasing the incidence of early pregnancy losses and negatively affecting fetal health [5–8].

Some studies conducted with specific groups of infertile women have assessed the association between infertility and thyroid autoimmunity alone or in association with other autoimmune diseases and the results have been conflicting. In a prospective study that compared 438 women with different infertility problems and 100 healthy parous women, it was found that the presence of antithyroid peroxidase (TPOAb) antibodies was significantly higher among women with female factor infertility when compared with controls [9]. On the other hand, in a study with 244 infertile women compared to 155 fertile women, there was no difference in the positivity of anti-TPO between groups [10]. Two recent reviews showed the pathogenesis of infertility and of the pregnancy losses associated with autoimmune disease [6, 7]. Despite the possibility that thyroid dysfunctions and autoimmunity may compromise the fertility of women of reproductive age, no specific studies have been carried out to measure the prevalence of infertility in groups of GD and HT.

The objective of the present study was to evaluate the prevalence of infertility periods higher than 12 months, in life of women with GD or HT followed in an Endocrinology service, and to determine possible associated factors.

## 2. Material and Methods

This cross-sectional study was conducted with two populations of women with GD and HT, followed up at the Endocrinology Clinic for Thyroid Autoimmune Diseases, School of Medical Sciences, University of Campinas (UNICAMP), São Paulo, Brazil. The project was conducted in accordance with the Declaration of Helsinki and was approved by the Institutional Review Board and all the women signed an informed consent form.

Fourteen women were excluded; 11 had lived sexually with a partner(s) less than 12 months and three had never started a sexually active life. The study only included women with a history of regular sexual relationship with a male partner for minimum period of 12 months. None of the women in the study had history of seeking treatment for infertility. All participants were interviewed by the same obstetrician-gynecologist, using a structured questionnaire specially made for the research, aiming to collect information about the previous reproductive life. The interviews were conducted from February 2011 to September 2012.

### 2.1. Subjects

The age requirement for admission to the study was 18–50 years for the GD group and 18–60 years for the HT group. Since women with HT are mostly followed up at primary healthcare level, in order to obtain the required sample size, the age limit for inclusion in the study was increased in this group. Women who reported a male factor of infertility and those with impaired cognition and/or access to past memories were excluded from the study.

### 2.2. Procedures

After the interview, the women's medical charts were analyzed to determine the characteristics of their disease, the date of their diagnosis, the time since diagnosis, and the results of laboratory and imaging tests and to confirm whether any associated autoimmune diseases had been diagnosed. The women were admitted sequentially to the study until the proposed number for each group was reached.

*Infertility* was defined as the absence of pregnancy in sexually active women having regular unprotected intercourse with a male partner for an exposure period of at least 12 months. Pregnancy losses were computed separately and corresponded to the sum of the number of miscarriages and stillbirths. The variables evaluated in both groups were sociodemographic factors, obstetric history, age at diagnosis, time of the disease (years between diagnosis and the date of the interview), presence and the degree of ophthalmopathy, the presence of antithyroid peroxidase (anti-TPO) and antithyroglobulin (anti-Tg) serum antibodies, presence of goiter, volume of the goiter, thyroid nodule, a history of primary ovarian insufficiency (POI), a history of any other autoimmune diseases, and a family history of autoimmune diseases.

### 2.3. Sample Size and Statistical Analysis

Based on the prevalence of women with hypothyroidism (3%) and hyperthyroidism (1%) [4], assuming a prevalence of infertility in the population of around 10% (1) and estimating that the prevalence of infertility in the study groups was the double (20%), with level of significance of 5% and a sampling error of 10%, the sample size was calculated at 61 subjects for the HT group. Since the ratio of prevalence between GD and HT is 3 : 1 and the sample size of Hashimoto's thyroiditis was 61 patients, the sample size for the DG group was estimated at 183 subjects.

For the statistical analysis, the Statistical Analysis System (SAS) software program, version 9.2, was used. Mann-Whitney test, chi-square test, and Fisher's exact test were used to compare all the variables in relation to the variable *infertility*. Wilcoxon test was used to compare the mean number of pregnancies prior to and after diagnosis and the Mann-Whitney test was used for the same comparison between the two age subgroups. Bivariate analysis was performed and the odds ratios (OR) were calculated with their respective 95% confidence intervals (95% CI) for the frequency of each variable. Next, multiple logistic regression was performed using the stepwise selection criteria to determine the multiple OR for the variable *infertility*. Additionally, the analysis was adjusted for the association between number of pregnancies prior to the diagnosis of GD or HT and multiple logistic regression to determine the multiple OR for the variable *infertility* was redone separately for the two subgroups of age at diagnosis for groups of GD or HT. Significance level was established at 5%.

## 3. Results

In the 193 women with GD and the 66 with HT enrolled in this study, the prevalence of infertility was 52.3% and 47.0%, respectively. The distribution of the groups (the women who reported infertility and those who did not, referred to from here on as the “*Infertility*” and “*No Infertility*” groups) is shown in Table 1.

In GD there were no significant differences in characteristics between *Infertility* and *No Infertility* groups. Concerning their medical history, one in 10 women had at least one associated autoimmune disease such as rheumatoid arthritis, systemic lupus erythematosus (SLE), vitiligo, and urticaria. In HT there was no significant difference between *Infertility* and *No Infertility* groups, except for the mean time of the disease, 5.5 ± 4.2 years and 9.1 ± 5.6 years, respectively, (*P* = 0.004) (Table 1). Regarding their medical history, 12.6% of the women in the *Infertility* group had at least one associated autoimmune disease such as rheumatoid arthritis, vitiligo, and urticaria.

There were no significant differences in the variables on pregnancy in the women with GT and HT in the *Infertility* and *No Infertility* groups (Table 2).

Analysis of the mean number of pregnancies prior to and after diagnosis of GD or HT is shown in Table 3 as a function of the woman's age at the time of diagnosis. Evaluation of the entire sample of women in both the GD and the HT groups shows that more pregnancies occurred before rather than after diagnosis of the disease (*P* < 0.001). Of all the women with GD, 84 (43.5%) were diagnosed with the disease prior to or at 35 years of age, while 27 of the women with HT (41%) were diagnosed at this age. More pregnancies occurred prior to diagnosis of the disease, both in the GD group and in the HT group, irrespective of the woman's age at the time of diagnosis (*P* < 0.001). The mean number of pregnancies was significantly lower in women who were 35 years old or younger at diagnosis when compared with those older than 35 (Table 3).

Analysis of the mean number of pregnancies prior to and following a diagnosis of GD or HT was done only for women aged 45 years or older (period of reproductive life ended) at the time of interview and the evaluation of women in both the GD (92 women) and the HT (39 women) groups shows that more pregnancies occurred before rather than after diagnosis of the disease (*P* < 0.001) (data not shown).

**Figure 1:**
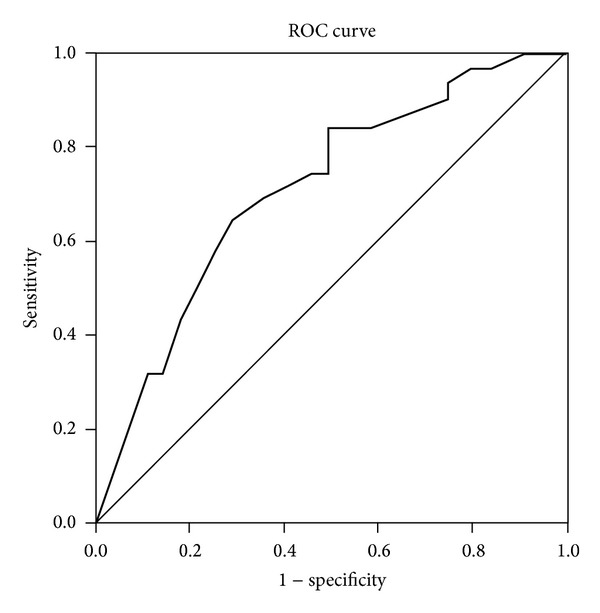
Receiver operating characteristic (ROC) curve for the variable *time of the disease* in the HT group, using infertility as the gold standard. AUC: 0.713; 95%CI: 0.5888–0.838; *P* = 0.003. Cut-off point: time of disease ≤6.5 years. Sensitivity 64.5%; specificity 71.4%.

Univariate analysis performed in the GD group showed that a moderate/severe degree of exophthalmos was indirectly associated with infertility (OR = 0.47; 95% CI: 0.22–0.99; *P* = 0.048). No other significant differences were found for any of the other variables evaluated (data not shown). The same analysis conducted in the women in the HT group showed an indirect association between infertility and time of the disease. The women who had been diagnosed with the disease more recently were five times more likely to have reported a period of infertility (*P* = 0.002) (data not shown). A receiver operating characteristic (ROC) curve was constructed for the variable *time of the disease* in the HT group, using infertility as the gold standard, and the area under the curve (AUC) was calculated together with the respective 95% CI (Figure 1). It was found that the optimal cut-off point for sensitivity (64.5%) and specificity (71.4%) was when the time of disease was ≤6.5 years. The variable *time of the disease* was then dichotomized into <6 years or ≥6 years.

Following multiple regression analysis and after adjusting for the association between the number of pregnancies prior to the diagnosis of GD or HT, the only variable found to be associated with infertility in the HT group was the time of the disease. A shorter time of the disease, especially less than six years, was associated with six times more chance to be infertile. The only variable found to be associated with infertility in the GD group was the age (Table 4).

Multiple regression analysis was performed by two subgroups of age at diagnosis, ≤35 years and >35 years of age. In the HT group the women with ≤35 years of age had the variable age indirectly associated with infertility, while in women aged >35 years the shorter time since diagnosis was associated with infertility. In the GD group, women aged ≤35 years had the variable time since diagnosis directly associated with infertility and none of the variables evaluated were found to be associated in the women with >35 years of age (Table 4).

## 4. Discussion

The prevalence of infertility, defined as the absence of pregnancy in sexually active women having regular unprotected intercourse with a male partner for an exposure period of at least 12 months, 52.3% in GD and 47.0% in HT, was high when compared to the figures reported for the general population, which have ranged from 7.4% to 24.0% in population-based studies [2, 3]. Age ≤ 35 years at diagnosis had an effect on the mean number of pregnancies in this group, which were around half that found for women who were over 35 years of age at the time of diagnosis. Furthermore, after the diagnosis of GD or HT, pregnancies were always fewer irrespective of the woman's age. It should be taking into account that in fertile couples, pregnancy rates of ~57% in the first three months of exposure and 81% after 12 months of exposure have been described [11]. Fertility rates range in accordance with the woman's age, with a decline beginning at 35 years of age, and women aged 45 years have a chance of getting pregnant close to zero [12]. A study with 2,193 women treated with intrauterine insemination of donor semen and separated according to age group (≤25 years, 26–30 years, 31–35 years, and >35 years) showed 12-month pregnancy rates of 73.0% for the first two groups, 61.0% for women of 31–35 years of age, and 54.0% for the women over 35 years of age [12].

Glandular dysfunction may be responsible for infertility. In GD group, after the adjusted association between number of pregnancies prior to the diagnosis the variable age was associated with the infertility, and in the subgroup with ≤35 years of age at diagnosis the variable time since diagnosis showed a positive association with infertility. Patients with GD generally require a longer period of clinical management in order to achieve a state of euthyroidism and this could explain a woman's persistent difficulty in conceiving.

On the other hand, in HT euthyroidism is more easily achieved and does not explain a longer period of infertility as a result of thyroid dysfunction. In the subgroup of women with ≤35 years age at diagnosis of HT, which showed lower mean of number of pregnancies, younger age was associated with infertility.

This result was consistent with that found after the analysis of the total sample of women in HT group when the only variable associated with infertility was a shorter time since diagnosis. Women who were diagnosed less than six years previously had a sixfold higher risk of infertility. There is no explanation for this finding. A study conducted to evaluate the effect of specific cellular immunity triggered by the TPO and Tg antigens concluded that these are both recognized by the CD8+ cells and are involved in the thyroid destruction process, leading to the clinical manifestation of HT. After the diagnosis, in the subsequent time periods of 1–5 years, 5–10 years, and >10 years of the disease, a progressive increase was found in TPO- and Tg-specific T cells in peripheral blood, with no correlation with circulating anti-TPO or anti-Tg antibodies [13]. Although cell immunity increases with the time of the disease observed in HT [13], it is possible that in the autoimmune process leading to glandular failure in the initial years, which is then a more exuberant process involving higher levels of circulating antibodies, the repercussions on the balance of the neuroendocrine mechanisms of reproduction may also be greater. Over time, with the substitution of the parenchyma by inflammatory infiltrate and fibrosis, and with the use of replacement therapy, the hormonal mechanisms of reproduction may come back into balance and fertility may return.

Also among women aged 45 years or older at the time of interview, which were considered to have completed their period of reproductive, more pregnancies before rather than after diagnosis of GD or HT were observed. Therefore, the decrease in the mean number of pregnancies following diagnosis in cases of both GD and HT found in this study suggests that the autoimmune mechanism may be responsible for this reduction in fertility.

The association between autoimmune thyroiditis, infertility, and/or successive pregnancy losses is a controversial issue [6, 14, 15]. One study evaluated women with and without autoimmune thyroiditis, all of whom were euthyroid during a cycle of in vitro fertilization, reported significantly lower rates of oocyte fertilization and grade A embryos in the 17 women who tested positive for anti-TPO and anti-Tg antibodies in serum and follicular fluid in relation to the controls; however, no differences were detected in positive pregnancy tests or early miscarriage rates [15].

The limitations of our study are those inherent to its design. Some of the variables were collected at the interviews and could not be confirmed, including data on pregnancy losses and family history of the diseases. With respect to pregnancy losses, were reported by around 20% of the women in the two groups, results which are comparable with the rates described in other studies [16, 17]. We have to take into account that the women in our study did not receive obstetric care at the same institution, and this could be a possible bias in the results; however, it is unlikely that women have been mistaken with respect to the number of births and miscarriages during their lives. Another limitation was the low statistical power (74%) for the comparison of the degree of exophthalmos between the groups with and without infertility. Perhaps the increased sample size could have found the possible interaction between infertility and severity of GD expressed by the degree of exophthalmos.

The prevalence of infertility among women with HT or GD was high, around 50%. This study confirmed the hypothesis that fertility is impaired in women with autoimmune thyroid disease, especially after the diagnosis of GD and HT; nevertheless, additional studies are needed to corroborate these findings.

## Figures and Tables

**Table 1:** Characteristics of the women with GD and HT who reported or did not report a period of infertility.

Characteristics	Infertility	No infertility	*P* value
*Graves' disease n* (%)	101 (52.3)	92 (47.7)	
Age^a^	44.3 (9.4)	42.2 (9.3)	NS^b^
Age at menarche (years)^a^	12.8 (1.8)	12.6 (1.6)	NS^b^
Age at sexual debut (years)^a^	17.6 (3.8)	17.5 (3.7)	NS^b^
Age at diagnosis (years)^a^	36.5 (9.8)	35.8 (9.1)	NS^b^
Time of the disease (years)^a^	7.8 (5.9)	6.3 (4.6)	NS^b^
Exophthalmos	57 (56.4)	60 (65.2)	NS^c^
Goiter volume (cm^3^)^a^	26.4 (22.2)	26.2 (18.9)	NS^a^
Thyroid nodule	28 (31.4)	25 (29.7)	NS^c^
Anti-TPO	87 (87.9)	72 (84.7)	NS^c^
Anti-Tg	61 (61.6)	52 (58.4)	NS^c^
Other autoimmune diseases	11 (10.9)	11 (11.9)	NS^c^
Family history of thyroid disease			
Graves' disease	14 (13.8)	14 (15.5)	NS^c^
Hashimoto's thyroiditis	13 (12.8)	7 (7.8)	NS^c^
Family history of other autoimmune diseases	47 (46.5)	38 (42.2)	NS^c^
*Hashimoto's thyroiditis n* (%)	31 (47.0)	35 (53.0)	
Age (years)^a^	44.7 (10.4)	46.2 (10.8)	NS^b^
Age at menarche (years)^a^	12.4 (2.0)	12.4 (1.6)	NS^b^
Age at sexual debut (years)^a^	18.5 (3.8)	17.4 (3.1)	NS^b^
Age at diagnosis (years)^a^	39.2 (10.6)	37.1 (11.4)	NS^b^
Time of the disease (years)^a^	5.5 (4.2)	9.1 (5.6)	0.004^b^
Goiter	10 (33.3)	13 (39.4)	NS^c^
Goiter volume (cm^3^)^a^	17.8 (22.3)	16.3 (11.8)	NS^b^
Thyroid nodule	15 (50.0)	10 (30.3)	NS^c^
Anti-TPO	27 (90.0)	33 (94.3)	NS^d^
Anti-Tg	27 (87.1)	29 (82.8)	NS^d^
Other autoimmune diseases	4 (12.9)	3 (8.6)	NS^d^
Family history of thyroid disease			
Graves' disease	2 (6.7)	5 (14.3)	NS^d^
Hashimoto's thyroiditis	11 (36.7)	10 (28.6)	NS^c^
Family history of other autoimmune diseases	18 (60.0)	18 (51.4)	NS^c^

“Infertility” and “No infertility” groups refer, respectively, to the group of women who reported a period of infertility and the group of women who did not.

NS: not significant; ^a^values consist of means ± standard deviation of the mean; ^b^Mann-Whitney test; ^c^chi-square test; ^d^Fisher's exact test; anti-TPO: antithyroid peroxidase antibodies; anti-Tg: antithyroglobulin antibodies.

**Table 2:** Pregnancy characteristics and primary ovarian insufficiency in the women with GD and HT who reported a period of infertility and those who did not.

History	Infertility	No infertility	*P* value
*Graves' disease n* (%)	101 (52.3)	92 (47.7)	
Number of pregnancies^a^	2.7 (1.4)	2.9 (1.6)	NS^b^
Number of living children^a^	2.4 (1.4)	2.4 (1.4)	NS^b^
Pregnancy losses (%)	19 (18.8)	20 (21.7)	NS^c^
Primary ovarian insufficiency (%)	8 (7.9)	8 (8.7)	NS^c^
*Hashimoto's thyroiditis n* (%)	31 (47.0)	35 (53.0)	
Number of pregnancies^a^	2.71 (1.53)	3.2 (1.8)	NS^b^
Number of living children^a^	2.26 (1.06)	2.9 (1.9)	NS^b^
Pregnancy losses (%)	7 (22.6)	7 (20.6)	NS^c^
Primary ovarian insufficiency (%)	4 (12.9)	2 (5.7)	NS^d^

NS: not significant; ^a^values are mean ± SD; ^b^Mann-Whitney test; ^c^chi-square test; ^d^Fisher's exact test.

**Table 3:** Comparative analysis of the mean number of pregnancies prior to and following diagnosis of GD and HT in accordance with the woman's age at diagnosis.

Number of pregnancies^a^	Woman's age				All women	
	≤35 years	*P* value	>35 years	*P* value	All age	*P* value
*Graves' disease *	(*n* = 84)		(*n* = 109)		(*n* = 193)	
Prior to diagnosis*	1.68 (1.41)	<0.001	3.07 (1.64)	<0.001	2.47 (1.69)	<0.001
Following diagnosis^#^	0.67 (0.96)		0.07 (0.3)		0.33 (0.73)	
*Hashimoto's thyroiditis *	(*n* = 27)		(*n* = 39)		(*n* = 66)	
Prior to diagnosis*	1.48 (1.31)	0.02	3.23 (2.01)	<0.001	2.52 (1.95)	<0.001
Following diagnosis^#^	0.67 (0.62)		0.03 (0.16)		0.29 (0.52)	

^a^All values are means ± SD. Wilcoxon test. Mann Whitney test for comparison between the two age subgroups: GD*(*P* value < 0.001), ^#^(*P* value < 0.001); HT*(*P* value < 0.001), ^#^(*P* value < 0.001).

**Table 4:** Factors associated with infertility in women with GD and HT, in the whole sample and the subgroups of age ≤35 years and >35 years, following multivariate logistic regression analysis.

Variable		OR (95% CI)	*P* value
*Hashimoto's thyroiditis *			
Time since diagnosis (years)	Continuous variable	0.84 (0.75–0.95)	0.007
Time since diagnosis	<6 years	6.17 (2.07–18.46)	0.001
	≥6 years	1.00	
Time since diagnosis (years)*	Continuous variable	0.83 (0.74–0.94)	0.004
Time since diagnosis*	<6 years	6.18 (2.03–18.75)	0.001
	≥6 years	1.00	
Age^#^	Continuous variable	0.77 (0.62–0.96)	0.025
Time since diagnosis^&^	<6 years	5.60 (1.36–23.06)	0.017
	≥6 years	1.00	
*Graves disease *			
Age*	Continuous variable	1.05 (1.01–1.09)	0.010
Time since diagnosis (years)^#^	Continuous variable	1.08 (1.01–1.17)	0.047

OR: odds ratio; 95% CI: 95% confidence interval. *Adjusted for the association between number of pregnancies prior the diagnosis. Stepwise variable selection criteria. ^#^Woman's age ≤35 years subgroup (*n* = 27 for HT group; *n* = 84 for GD group). ^&^Woman's age >35 years subgroup (*n* = 39 for HT group; *n* = 109 for GD group). Variables studied: age, number of pregnancies and deliveries, total number of living children, total number of premature infants, total number of pregnancy losses, pregnancies prior to and following diagnosis, pregnancy losses prior to and following diagnosis, complications in pregnancy prior to and following diagnosis, age at diagnosis, time since diagnosis, anti-TPO antibodies, anti-Tg antibodies, goiter, volume of goiter, thyroid nodule, history of POI, history of other autoimmune diseases, and family history of thyroid disease.
